# Otolith shape and microchemistry reveal fine-scale population connectivity in the myctophid *Benthosema glaciale* (Reinhardt, 1837) along a complex seascape

**DOI:** 10.1038/s41598-026-46216-3

**Published:** 2026-04-01

**Authors:** Francesco Saltalamacchia, Natalya D. Gallo, Karin Limburg, Arild Folkvord, Anne Gro Vea Salvanes

**Affiliations:** 1 Department of Biological Sciences, University of Bergen, Bergen, Norway; 2 Bjerknes Centre for Climate Research, Bergen, Norway; 3 Norwegian Research Centre (NORCE), Bergen, Norway; 4 Department of Environmental Biology, State University of New York College of Environmental Science and Forestry, Syracuse, New York USA; 5 Department of Aquatic Resources, Swedish University of Agricultural Sciences, Uppsala, Sweden; 6 Institute of Marine Research (IMR), Bergen, Norway

**Keywords:** Coastal populations, Fjord, Lanternfish, Mesopelagic, Oceanographic connectivity, Population structure, Ecology, Ecology, Ocean sciences

## Abstract

Myctophid fishes constitute a major component of the world’s ocean biomass and are seen as a potential resource for commercial exploitation. Within this family, the glacier lanternfish *Benthosema glaciale* (Reinhardt, 1837) is the dominant North Atlantic species and plays a key role in pelagic food webs in open waters, deep coastal regions and fjords. Previous studies employed genetic markers to evaluate population structuring across the species’ broad distribution range, detecting heterogeneity in shallow-silled fjords. However, genetic methods cannot reconstruct ontogenetically determined movements, and research on alternative methods is lacking. Here, we examine whether otolith features can resolve coastal connectivity in *B. glaciale*. We collected individuals from a coastal site and a range of fjord types along the complex Norwegian west coast to capture topographic and environmental characteristics that could give rise to population structuring. We show that otolith shape coefficients and temporally-resolved microchemical signatures reveal consistent and significant patterns of variation supporting the presence of subpopulations within small geographic distances. Contrary to earlier hypotheses, we propose that oceanographic dynamics affecting early life stages have a greater influence on shaping coastal mesopelagic populations than topographic barriers. This study presents the first evidence of the effectiveness of otolith morphometrics and microchemical analysis in addressing ecological questions in this abundant myctophid, and highlights their potential to provide valuable information for future management and conservation strategies.

**Supplementary Information:**

The online version contains supplementary material available at 10.1038/s41598-026-46216-3.

## Introduction

Mesopelagic fishes constitute the most abundant group of vertebrates on Earth^1^ and the estimated magnitude of their biomass has elicited international interest in the prospect of commercial exploitation^2^. Nonetheless, the ocean’s mesopelagic depths are expected to undergo widespread environmental changes, potentially leading to shifts in distributions and community structures^3–5^. Sustainable harvesting depends on well-known life history traits^6,7^. However, considerable knowledge gaps in the biology and ecology of mesopelagic fishes persist^7^, including basic information about population dynamics and connectivity^8,9^.

Biological connectivity reflects the genetic exchange between populations caused by movements across a species’ distribution range, and is an essential factor driving life histories and resilience to environmental change and commercial exploitation^10^. In marine fish, the degree of connectivity is linked to age-dependent vertical and horizontal migratory patterns^11^. During early life stages, advection and diffusion are the main dispersal mechanisms, often relying on the vertical distribution of fish larvae within advective layers subjected to wind and tidal forces^12^. After metamorphosis, population connectivity in pelagic species can be maintained by horizontal swimming across adjacent areas and, in some cases, by directed spawning migrations. Regional and local features of the seascape (currents, stratification, topography) affect both the spatial scale at which subpopulations are connected and the proportion of migrating adults and early life stages that are advected to other habitats^13^. Changes in current patterns, for instance, can alter dispersal routes, potentially reducing connectivity between subpopulations and impeding the replenishment of exploited stocks.

Additionally, topographical barriers such as continental shelves^14^ and shallow sills at the entrance of fjords^15^, as well as variations in water density along the coast^12^ can affect dispersal pathways of deep-dwelling species whose populations extend into inland basins. Understanding the spatial structure of marine populations, therefore, requires characterising their patterns of connectivity and life history variations^16^. Such knowledge is also relevant for assessing the potential impacts of climate stressors and will inform management authorities in their work on conservation strategies^17^.

Genetic markers^18^ and artificial tags^19^ are common tools for studying connectivity in marine species. However, genetic techniques cannot be used to determine whether individual exchange between basins occurs by advective transport of early life stages or by horizontal movement after metamorphosis. Another limitation of these methods is that even low levels of interbreeding can remove genetic differences^20^. Furthermore, experiments using external tags are not feasible for small schooling myctophids. However, bony fishes are equipped with natural tags in the form of otoliths.

The study of fish earstones, or otoliths, has seen a great increase in attention over the past decades^21–23^. Otoliths are composed mostly of calcium carbonate embedded in an organic matrix, plus a fraction (< 1%) of trace elements^24^. As metabolically inert structures, otoliths grow continuously throughout the organism’s life, proportionally to body size and without reabsorption^25^. The formation of seasonal growth zones allows age determination and, when the time of death is known, the dating of each portion of the otolith.

The morphological characteristics of fish otoliths are thought to reflect adaptive requirements related to their physiological functions and, as such, vary between species. At the same time, otolith shape arises from their accretionary growth, with material deposited incrementally along the otolith margin. Localised variation in growth rate along specific edges can therefore generate pronounced differences in overall morphology^26^. These growth dynamics are influenced by both genetically regulated developmental processes and environmentally mediated factors such as temperature, dissolved oxygen concentration, salinity, diet and somatic growth^27–30^. Consequently, multivariate descriptors of otolith shape may capture distinct sources of variation, with some axes reflecting heritable differentiation among biological units and others reflecting environmental gradients experienced during life^30^. Similarly, the deposition rates of different trace elements in the otolith matrix vary within species, with some elements (such as strontium and barium) thought to relate predominantly to water concentrations or environmental conditions, and others (e.g. phosphorus, magnesium) relating to physiological processes such as growth and reproduction^24^. Due to these characteristics, otolith shape and microchemistry have been successfully used to identify population structure^31,32^, uncover natal origin^33,34^, and track individual exchange between habitats^35,36^.

The glacier lanternfish *Benthosema glaciale* (Reinhardt, 1837), Myctophidae, is one of the dominant mesopelagic fish species in the North Atlantic Ocean^37^. Genetic studies from the Norwegian west coast^15,38,39^ identified a relatively homogenous population across a few deep-silled fjords and two semi-isolated genetic components in shallow-silled fjords. In deep-dwelling species occupying coastal waters, restricted exchange due to physical or hydrographic barriers and environmental heterogeneity limiting the movement of individuals can result in partial isolation and reduced gene flow^40^, leading to spatial structuring over relatively short distances^41^. It is hypothesised that shallow topographies such as fjord sills increase the local retention of planktonic life stages and prevent post-metamorphosis horizontal movements^15^. However, these hypotheses have not been tested on *B. glaciale*.

The availability of a genetically validated population structure for *B. glaciale* provides a strong baseline for assessing whether non-genetic features such as otolith shape and microchemistry can be used to elucidate connectivity patterns in this important mesopelagic species. Additionally, we aim to assess whether the elemental signature recorded in the otolith matrix can provide insights beyond what genetic markers offer, such as evaluating the timing of movements across shallow topographic structures.

We collected individuals from a range of fjord types across the Norwegian west coast (e.g. far inland versus close to the coast, shallow sills (<130 m) versus deep sills, shallow (<300 m) versus deep basins) to capture topographic and environmental characteristics that could give rise to population structuring. We hypothesise that: (i) otolith shape variation in *B. glaciale* increases with genetic distinctness and environmental differences, and can be used to assess population structure and the degree of connectivity between basins; (ii) similarly, considering the high environmental heterogeneity across the sampled region^42^, discrete biological units with limited connectivity will be characterised by different “otolith elemental fingerprints”; (iii) if shallow sills prevent horizontal movement, the chemical fingerprint of otolith increments formed during the juvenile stage (when individuals occur shallower in the water column) will differ between adjacent fjords after the initial phase of growth during which the maternal signature dominate the signal^43^. Conversely, no difference in juvenile microchemistry between two adjacent basins suggests a connection between the two areas at the time of formation of the growth increment, while increasing variation with age might signify progressive isolation.

## Methods

### Ethics declaration

This study reports findings on wild *Benthosema glaciale* caught by trawling onboard research vessels operated by the Norwegian Institute of Marine Research (Bergen, Norway). Sampling was carried out under permits granted by the Norwegian Directorate of Fisheries (Fiskeridirektoratet) for scientific purposes (Ref Nos. 2022/16403 - 2022/86673). Small mesopelagic fish, like our study species, die quickly in the trawl before reaching the deck. Our samples, therefore, consisted of deceased individuals, which were rapidly sorted and frozen. This research did not involve experimental work on live vertebrates. As such, the ARRIVE guidelines are not applicable to this study.

### Study species

*B. glaciale* is a small-bodied, short-lived fish widely distributed from the Baffin Bay and the Barents Sea^38^ down to the upwelling regions off the north-west African coast, including a relic population in the Mediterranean Sea^44^. Quintela and colleagues^38^ evaluated the species’ genetic population structure across most of its distribution range, identifying three major regional populations: the Mediterranean Sea, the North Atlantic Ocean, and deep west Norwegian fjords. Along Western Norway, individuals live up to 5-6 years and reach sexual maturity around age 2, or at a body size greater than 45 mm^45^. During daytime, *B. glaciale* occur mostly at depths greater than 200 meters and, at dusk, ascend from the mesopelagic to the epipelagic zone. Their vertical distribution follows a trend of increasing size with depth^46^ and, as a visual feeder, the species’ depth preference is associated with a ”light comfort zone”^47^.

### Study region and sample collection

**Fig. 1:**
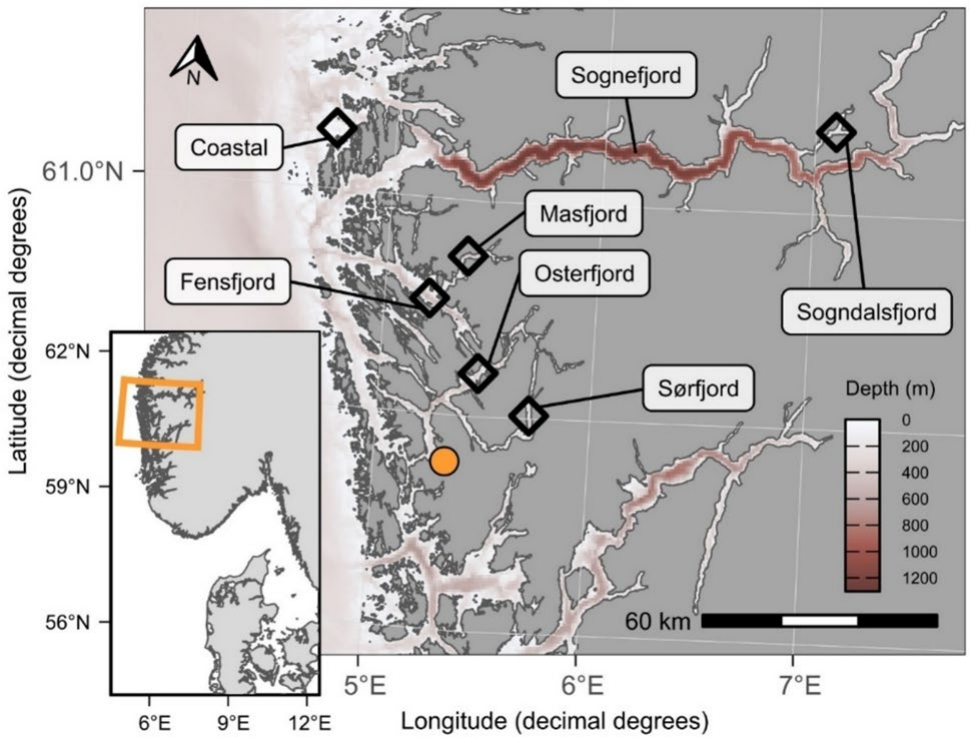
Sampling area along the Norwegian west coast for the study of otolith shape and microchemistry of *Benthosema* glaciale. The sampling sites are indicated by open diamonds on the main map: Coastal (abbreviated to COA throughout the manuscript), Fensfjord (FEN), Masfjord (MAS), Osterfjord (OST), Sogndalsfjord (SOG), Sørfjord (SØR). The location of the city of Bergen is highlighted by a circle for orientation. The map was produced in R v. 4.3.0^61^ using the *ggOceanMaps* package v. 2.1.1^62^.

*B. glaciale* were sampled in 2021 and 2022 from five fjords and a coastal location along the Norwegian west coast (Fig. 1). Sogndalsfjord (hereafter: SOG), with a maximum depth of 260 m, is a 21 km long basin branching out over a 25 m deep sill^48^ from the inner part of Norway’s longest and deepest fjord, Sognefjord. Our coastal sampling site (COA), with a bottom depth of 380 m, is located just north of Sognefjord’s mouth, east of the Norwegian shelf. About 30 km south lies Fensfjord (FEN), with a maximum depth of 680 m and in direct connection with the coastal waters through a 380 m deep sill^49^. Masfjord (MAS), 490 m at its deepest point, branches out of Fensfjord over a shallow sill at 75 m depth^50^. Osterfjord (OST) and Sørfjord (SØR) are part of a large estuarine system east of the city of Bergen, whose connection to the outer coast is restricted by a network of narrow and silled sounds^51^. The two fjords, with maximum depths of 650 and 400 m respectively, are adjoined at both ends but separated by sills. The deepest sill, at the southern connection, is located at a depth of 160 m^51^.

The combination of sill depths, surrounding bedrock types^52^, watershed size relative to basin surface area, and coastal proximity (Supplementary Table S1) differs across sites. Sill depth controls the extent of communication between the basin water of a fjord and the outer coast, affecting stratification^53^. Bedrock origin and the ratio of catchment to surface area are fundamental factors governing water and sediment chemistry^54,55^. The sampling sites also vary in their dissolved oxygen content: those closer to the outer coast (COA and FEN) maintain consistent ventilation, while oxygen levels decrease progressively from OST to MAS, SØR, and SOG (Supplementary Figure S5)^56^. As diel vertical migrators, lanternfishes regularly encounter different water masses during their movements. Light absorption patterns vary with oxygen content and salinity^57^ and, with bottom depth, constrain the vertical extent of the mesopelagic zone. These factors influence the range of ambient light available to vertically migrating fish and, consequently, the depth at which individuals can encounter their light comfort zone^58^. Despite the relatively small spatial extent of the study region, previous knowledge and our observations imply distinct environmental conditions across sites, providing a robust framework for interpreting connectivity based on chemical signatures.

Among the sampled basins, some have been previously surveyed for genetic studies of *B. glaciale*, with distinct subpopulations compared to coastal areas reported in MAS^15,39^ and OST^38^. This differentiation has been hypothesised to stem from the shallow sills isolating these basins. In the case of MAS, it is hypothesised that the sill depth is low enough to hinder the passage of larvae^15^. However, no samples from the adjacent fjords on the other side of these sills have been examined. Based on these previous works, we expect MAS and OST to differ from the coastal site and from each other. Furthermore, if shallow sills are the main driver of population structuring in the region^15,38^, we expect our samples from MAS, the OST/SØR system and SOG (Fig. 1) to constitute three distinct units, and COA and the deep-silled FEN to be homogeneous.

In FEN, MAS, OST and SØR, we sampled the water column between 200 and 300 m depth, mainly at night, using a Harstad trawl fitted with a Multisampler (an attachment allowing opening and closing of the codends at chosen depths)^59^. Additional daytime Harstad trawls were used to supply catch from 50-150 m above the seabed. At the shallower sites (COA and SOG), *B. glaciale* was sampled by bottom trawling at night. All hauls were processed based on the protocol described in Salvanes et al.^60^. Random subsamples from each catch were frozen for otolith extraction.

### Otolith extraction, imaging, and age reading

**Fig. 2:**
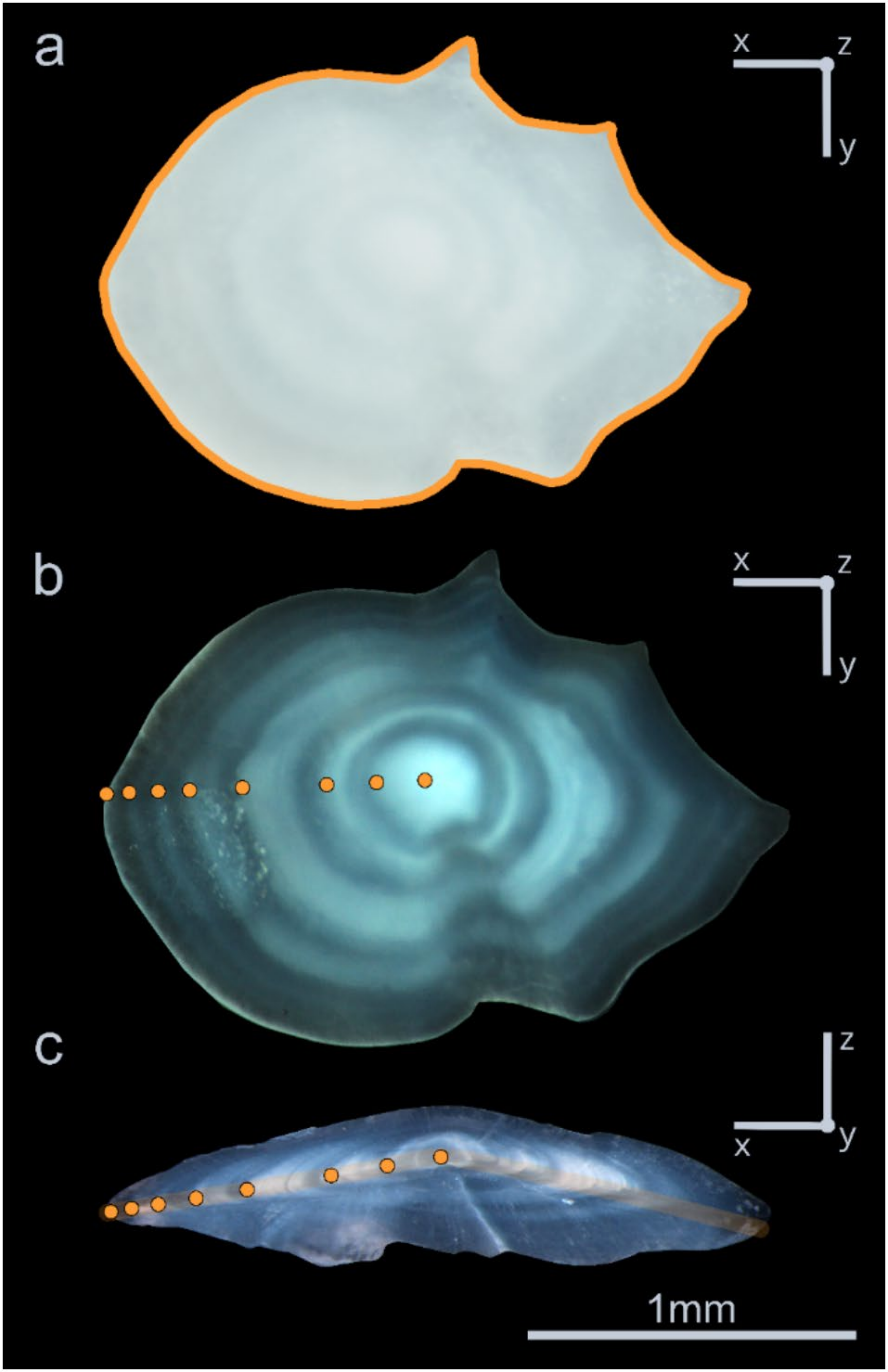
Annotated views of a left otolith of *B.* glaciale caught in Masfjord, Norway. (**a**) “Dry” top view of the distal side (used for shape analysis): otolith edge highlighted. (**b**) “Wet” top view (used for ageing): the circles follow the preferred direction of ageing towards the dorsal margin, and highlight the same landmarks represented in c. (**c**) Longitudinal section (used for microchemistry analysis): the laser path is highlighted as a shaded line. Only the portion of the otolith between the core and the dorsal margin (left side of the picture) was used for analyses. Three-dimensional Cartesian coordinate axes are given on the right side of each panel to illustrate the sectioning direction (x: dorso-ventral axis; y: antero-posterior axis; z: transverse axis)*.*

Individuals from each sampling site were randomly selected from the frozen samples for further analysis. If fewer than 60 individuals were available, we processed all. 100-130 individuals each were thawed from FEN and MAS, from which a larger sample was available due to the high scientific interest in this area. After thawing, standard length (SL) was measured to the nearest millimetre. Sagitta otoliths (i.e. the larger of the three pairs of otoliths in bony fishes) were extracted under a dissection microscope and rinsed in distilled water. All otoliths (left and right for each fish) were left to dry, then placed sulcus down on a dark tray and individually photographed for otolith shape analysis, ensuring good contrast against the background (Fig. 2a). Each otolith pair was then covered with a drop of distilled water to enhance the visibility of the annuli and photographed again for age reading (Fig. 2b). All pictures were taken under reflected light. To obtain a conversion factor from pixels to millimetres, a picture of a micrometre calibration slide was taken at the beginning of each photographing session or whenever a change in magnification was required. The study was performed only on otoliths that did not show any signs of damage or vaterite formation^63^. Age determination was performed on the pictures of wet otoliths by inspecting the annuli from both otoliths in a pair, under the assumed birth date of 1^st^ January. The preferred direction of ageing was along the longest Feret axis, extending from the centre of the otolith to the dorsal margin (Fig. 2b).

All data processing and analysis were performed in R v. 4.3.0^61^. Throughout the study, statistical analyses follow the protocol described in^64,65^.

### Otolith shape analysis

#### Data processing

Outline detection and shape coefficients (providing a numerical reconstruction of otolith shape) were obtained from the pictures of dry otoliths (Fig. 2a) following the method described by Libungan & Pálsson^66^. All the images of left-side otoliths were flipped horizontally to align with the direction of the right otoliths. Otolith outlines were elaborated using the *shapeR* package v. 1.0.1^66^. Each outline was overlaid on the original image and visually evaluated for good accuracy along the edge of the otolith. Whenever an outline was found to be inaccurate, the image was processed again with a different threshold value or edited in GIMP (GNU Image Manipulation Program) v. 2.8.22 to enhance contrast. All outlines were smoothed to reduce pixel noise. To correct for the effect of size, rotation, and positioning, the outlines were automatically scaled to the same area, centred on their centroid, and oriented horizontally along the largest Feret axis. Sixty-three independent shape coefficients from radii drawn at constant angular intervals from the otolith centroid to the outline were then obtained by Discrete Wavelet Transformation using Daubechies least-asymmetric wavelets. An additional set of morphological indices was also calculated: otolith ellipticity, rectangularity, roundness^67^, circularity and crenulation index^68^. Throughout the study, “shape coefficients” will refer collectively to Wavelet coefficients and morphological indices. To minimise the impact of ontogenetic effects on otolith shape, we restricted our analyses to individuals aged between 2 and 5 at capture (Table 1).

**Table 1:** Summary of the samples included in the study of the otolith shape of *B. glaciale*.

Site	Abbreviation	Sampling year	N	SL ± SD	AAC ± SD
Coastal	COA	2022	19	84.21 ± 6.18	4.68 ± 0.48
Fensfjord	FEN	2021	86	60.40 ± 7.27	3.73 ± 0.82
Masfjord	MAS	2021	124	54.81 ± 7.93	3.11 ± 1.08
Osterfjord	OST	2021	20	44.70 ± 6.29	3.05 ± 0.89
Sogndalsfjord	SOG	2022	10	58.70 ± 9.14	2.53 ± 0.50
Sørfjord	SØR	2021	53	48.62 ± 6.28	3.77 ± 0.97
Total			312	56.57 ± 11.18	3.47 ± 1.05

Site: sampling location. Abbreviation: short notation for each site, adopted throughout the manuscript. N: number of individuals. Standard length (SL, mm) and age at capture (AAC, years) are reported as mean ± standard deviation (SD) for each site and overall.

#### Statistical analysis

To account for allometric relationships between otolith shape and body size, the shape coefficients were tested for interactions between sampling site and SL using Analysis of Covariance (ANCOVA) with Bonferroni correction of the p-values. All coefficients showing a significant interaction (p > 0.05) were discarded; those significantly correlated with SL were corrected using regression-based standardisation^69^. This process involved two steps. First, we applied a within-group correction to remove any effect of body size on otolith shape within each sampling site. Second, as data exploration revealed significant differences in SL between sites, we applied a common-slope correction to account for the possibility of poorly overlapping length distributions. All coefficients unrelated to SL were left unchanged.

In otolith morphometric studies, it is common to use only otoliths from one side of the head (left or right) or replace a missing otolith with its counterpart from the opposite side. This may introduce bias in populations exhibiting bilateral asymmetry in otolith shape^70^. As the main focus of this study was not to evaluate the direction and magnitude of directional asymmetry, but rather to account for it while addressing our research question about population structure, our chosen approach to test for differences between left and right-side otoliths was based on a modified version of the multivariate methodology described in Mahé et al.^70^ (further description provided in Supplementary information S1).

The multivariate mixed-effects model revealed no significant effect of standard length (SL) on the shape coefficients (Supplementary Table S2), confirming that the standardisation was effective and that differences in body size did not confound any remaining shape variation. However, we found that both the side (left-right otolith) and the sampling site had significant effects, along with their interaction (Supplementary Table S2).

Given our primary interest in understanding geographical variation among sites, we proceeded by applying a redundancy analysis (RDA, from the *vegan* package^71^) conditioned by side to the matrix of shape coefficients. This allowed the compression of the multivariate space into fewer dimensions (canonical components, CCs) while avoiding collinearity. We then assessed shape variation across the study region (assumed to reflect population structuring) by examining the position of each site’s centroid on a biplot of the first two CCs. After checking for homogeneity of variance, the mean scores of each site across all CCs were used as input for a Ward’s hierarchical agglomerative algorithm to generate a dendrogram based on squared Euclidean distances. Permutational analysis of variance (PERMANOVA) with 2000 permutations, also based on Euclidean distances, was used to perform pairwise comparisons between sites, allowing the assessment of significance under the null hypothesis of no differences among groups. To account for an increased probability of Type I errors due to multiple testing, a Benjamini-Hochberg correction (BH) was applied to the p-values.

Waterway distances (in kilometres) between sampling sites were computed using the *raster* package v. 3.6.32^72^. Mantel tests were then used to assess the correlation between pairwise distances in otolith shape and geographic distances between sites.

### Otolith microchemistry

#### Measurement of trace element concentrations

A subset of otoliths from the larger individuals from each year and fjord was selected for processing by Laser Ablation Inductively Coupled Plasma Mass Spectrometry (LA-ICPMS) (Table 2). Due to LA-ICPMS techniques being partially destructive, only one otolith per fish could be sampled for this analysis. To our knowledge, asymmetry in elemental signatures within otolith pairs has not been reported outside species characterised by dorso-ventral body-shape asymmetry. However, we preferred not picking one side arbitrarily, but rather sampled the left or right otolith from each individual using a random binary number generator, with each side having an equal chance of being chosen. The selected otoliths were embedded in a two-component cold-setting epoxy resin. The otoliths were sectioned in a longitudinal plane passing through the core with a precision low-speed saw equipped with 0.3 mm diamond wafering blades and a 1-mm separator. The sections were manually polished to expose the core (Fig. 2c) with 1000 to 2500-grit silicon carbide grinding paper, washed in distilled water, and mounted on microscope slides.

**Table 2:** Summary of the samples included in the study of otolith microchemistry of *B. glaciale*.

Site	Abbreviation	Age	Year of increment formation	N
Coastal	COA	1	2014-2018	6
		2	2015-2019	6
		3	2016-2020	6
Fensfjord	FEN	1	2016-2018	11
		2	2017-2019	11
		3	2018-2020	10
Masfjord	MAS	1	2016-2018	13
		2	2017-2019	12
		3	2018-2020	12
Osterfjord	OST	1	2015, 2017-2019	8
		2	2016, 2018-2020	8
		3	2017, 2019-2020	7
Sogndalsfjord	SOG	1	2017-2020	6
		2	2018-2021	6
		3	2019-2021	5
Sørfjord	SØR	1	2016-2017	8
		2	2017-2018	8
		3	2018-2019	8

Site: sampling location. Abbreviation: short notation for each site, adopted throughout the manuscript. Age: estimated age at otolith increment formation. Year of increment formation: years at which the individual increments were deposited. N: number of individual increments for each site/age.

Otolith trace elements were sampled at the College of Environmental Science and Forestry of the State University of New York (SUNY-ESF) in Syracuse, New York (USA). We measured a suite of fifteen potentially informative elements based on previously published work ^24,73^: lithium, boron, sodium, magnesium, phosphorus, calcium, chromium, manganese, iron, cobalt, copper, zinc, strontium, barium and lead. The isotopes used were: ^7^Li, ^11^B, ^23^Na, ^25^Mg, ^31^P, ^43^Ca, ^53^Cr, ^55^Mn, ^57^Fe, ^59^Co, ^63^Cu, ^66^Zn, ^88^Sr, ^138^Ba, ^208^Pb (from here on, mass numbers will be omitted in the text). Elemental concentrations were collected with a triple quadrupole ICP-MS equipped with an excimer laser (193 nm wavelength). Instrument control and data acquisition were managed through Qtegra software v.2.14 (Thermo Fisher Scientific). Edge-to-edge transects passing through the otolith core were defined along the major growth axis (Fig. 2c). Spot transects were ablated along the selected paths (Fig. 2c) following a pre-ablation to sanitise the surface. Laser settings and session setup are detailed in Supplementary Information S2.

After ablation, the otolith sections were covered with a drop of glycerol to ensure good visualisation of the laser path and the underlying otolith macrostructure, and photographed (Fig. 2c) under reflected light.

#### Data processing

A semi-quantitative data reduction was performed with the software Iolite v. 4.10.1 (Elemental Scientific Lasers) following the protocol described in Paton et al.^74^. Further processing and analysis were performed in R.

In three of the sampled elements (Pb, Co, Cr), more than 20% of measurements were below the limit of detection (LOD). We therefore proceeded to further analyses retaining only Li, Na, Mg, P, Ca, Fe, Sr, Ba, B, Mn, Cu and Zn. All below-LOD and outlier measurements (4% and 0.4% of the total, respectively) across the retained elements were corrected by linearly interpolating between the two closest acceptable measurements on each side. Finally, all analytes were expressed as [element (ppm)]/[Ca (ppm)]*10^5^. Details on data reduction and the definition of LODs and outliers can be found in Supplementary Information S3.

#### Matching microchemistry data to individual life span

Annual growth increments along the laser path were measured in ImageJ v.1.52p^75^ from the post-ablation pictures. Measurements were taken from the centre of the otolith core to the dorsal margin (Fig. 2c). We did not measure landmarks along the entire transect, as seasonal zones occur symmetrically around the core, and the dorsal direction was clearer to read. The number of zones was validated by comparing them with age readings from whole otoliths (Fig. 2b, c). The distance (μm) between laser ablation points (average time gap (s) times laser speed (μm·s⁻1)) was used to assign each point to a specific age at formation (and corresponding year) based on the measured annual growth increments. As our main interest was to evaluate connectivity across shallow topographies, we retained only trace element concentrations corresponding to ages 1 (therefore excluding earlier maternal effects) to 3 (assumed to represent the first year after sexual maturation).

### Statistical analysis

All elemental concentrations were normalised to a mean of 0 and a standard deviation of 1, and averaged within each individual annual increment.

Symmetry in the chemical signature between left and right otoliths was assessed at locations from which microchemical data were available from at least four left and four right otoliths (FEN, MAS, SØR) using PERMANOVA (α = 0.05) (Supplementary table S3). The observed lack of significant differences within locations supported the decision to combine left and right otoliths for subsequent analyses.

Due to the nature of the data, the otoliths sampled at each site originated from fish spanning multiple cohorts. Consequently, the increments available within each area were deposited over more than one year. We therefore limited our analyses to a specific range of cohorts (2013-2019, 2 to 5 cohorts per site; Table 2, Supplementary Table S4), pooled within each site to ensure a good balance between maintaining sufficient sample size for statistical analysis and minimising potential confounding effects caused by temporal variability in the microchemical signal. To establish a benchmark for testing the robustness of our results (i.e., excluding a cohort effect), we conducted a preliminary analysis that included only sites with at least four individuals from the most represented cohort (2016). These were FEN, MAS, and SØR. Since more than four individuals were also available from the 2017 FEN cohort, this group was also included. We hypothesised that, with a stable signal, the between-site variation identified by pairwise comparisons among these four groups (PERMANOVA, α = 0.05) would align with the results obtained when pooling all cohorts. Furthermore, the increments deposited across two cohorts within the same site (i.e., FEN 2016 and FEN 2017) would not differ significantly.

Multivariate analysis of trace element variation across the study region was carried out in a similar way as for the shape coefficients. First, we ran separate RDAs on the matrix of elemental concentrations for increments deposited at ages 1 to 3. The canonical scores were tested for overall significance (α = 0.05) using ANOVA-like permutation tests with 2000 permutations. PERMANOVA with BH correction of p-values (α = 0.05) was then adopted to test for the significance of trace element variation among each pair of sites. Finally, the correlation between pairwise distance in trace element composition and geographic distance was tested using Mantel tests.

## Results

### Otolith shape analysis

**Fig. 3:**
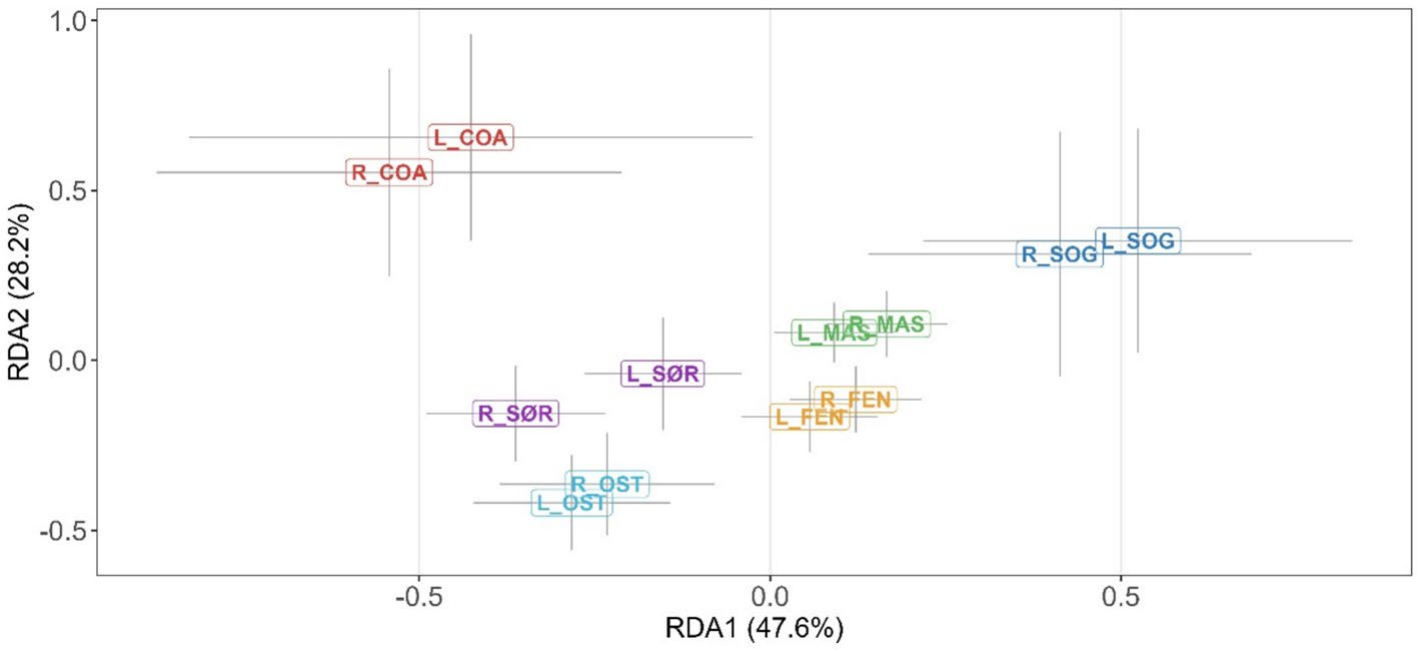
Redundancy Analysis (RDA) ordination diagram representing otolith shape variation of *B. glaciale* between selected locations across the west Norwegian coast. Labels and bars represent the site centroid and standard errors of the canonical scores based on otolith shape coefficients. The RDA was conditioned to remove the difference between the left and right otoliths, which was of no interest to this study. L and R prefixes indicate left and right otoliths, respectively. Colour coding representing each sampling site is provided for clarity. Site labels: COA (Coastal), FEN (Fensfjord), MAS (Masfjord), OST (Osterfjord), SOG (Sogndalsfjord), SØR (Sørfjord).

A distinct pattern emerged from the biplot of the first two canonical components (CCs) from the otolith shape analysis (Fig. 3). The site centroids for the coastal location (COA) and Sogndalsfjord (SOG) stand in stark contrast, positioned at opposite ends of the first CC, which accounts for approximately 48% of the explained variance. Along this axis, the standard errors for the centroids of Fensfjord (FEN) and Masfjord (MAS) exhibit substantial overlap, while those of Osterfjord (OST) and Sørfjord (SØR) overlap only partially. The second CC captures an additional 28% of the explained variance, with the northernmost samples (COA and SOG) showing higher scores compared to the fjords in the southern part of the study area. Additionally, along this axis, MAS and FEN do not overlap, and SØR and OST show reduced overlap compared to the first CC.

Similar patterns of otolith shape variation between left and right otoliths across sampling sites were revealed by side-conditioned RDA. This indicates that, despite the inherent bilateral asymmetry, the factors driving spatial differentiation among sites are robust and can be generalised across both sides. Consequently, we focused our subsequent analyses exclusively on the left-side otoliths.

**Fig. 4:**
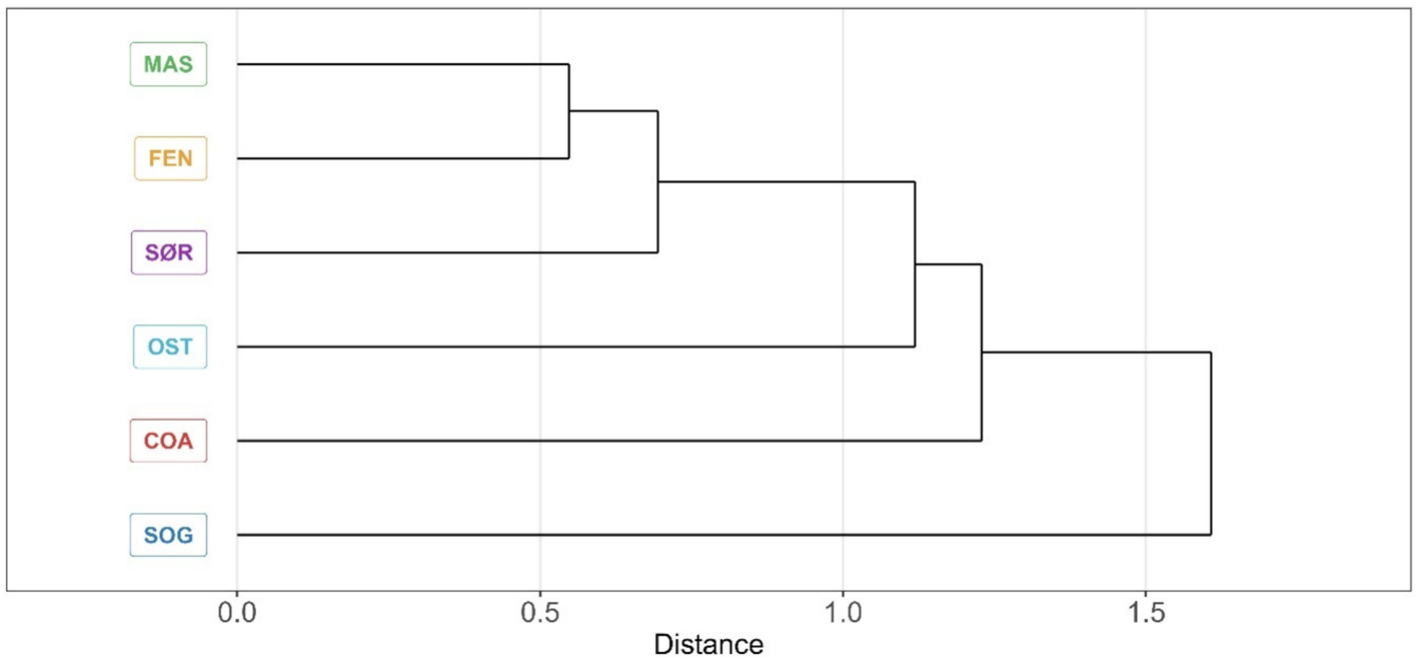
Dendrogram of otolith shape relationships of *B. glaciale* across six sampling sites along the Norwegian west coast, generated by Ward’s hierarchical agglomerative algorithm using squared Euclidean distances. The analysis was conducted on left-side otoliths, as the pattern of differentiation between sites was consistent for left and right otoliths.

The two neighbouring sites FEN and MAS exhibit the most similar otolith morphology: in the hierarchical cluster analysis, these sites had the shortest distance between their cluster centroids, based on mean canonical scores from the RDA of the left otoliths (Fig. 4). Notably, the samples from the four southern fjords display closer relationships with one another compared to COA and SOG, the latter appearing to be the most distinct site overall.

Otolith shape differs significantly among nearly all sampling sites, as supported by pairwise comparisons of canonical scores (PERMANOVA, BH α = 0.05), with FEN and MAS being the only pair without a significant difference (Table 3). Geographically distant sites tend to show greater differences in otolith shape, as reflected by a strong positive correlation (r = 0.707) that just exceeded the significance threshold (p = 0.053) in the Mantel test (Supplementary Fig. S1).

**Table 3:** Statistical significance (after Benjamini–Hochberg correction) of pairwise comparisons based on Permutational Analysis of Variance (PERMANOVA, Euclidean distances with 2000 permutations) representing differences in otolith shape of *B. glaciale* sampled in six sites along the west Norwegian coast.

	COA	FEN	MAS	OST	SOG
FEN	0.001 **				
MAS	0.002 **	0.119			
OST	0.002 **	0.002 **	0.002 **		
SOG	0.003 **	0.007 **	0.019 *	0.002 **	
SØR	0.003 **	0.046 *	0.036 *	0.008 **	0.003 **

Bold values highlight statistical significance (p < 0.05). Symbols: .<0.10, *<0.05; **<0.01, ***<0.001.

### Otolith microchemistry

**Fig. 5:**
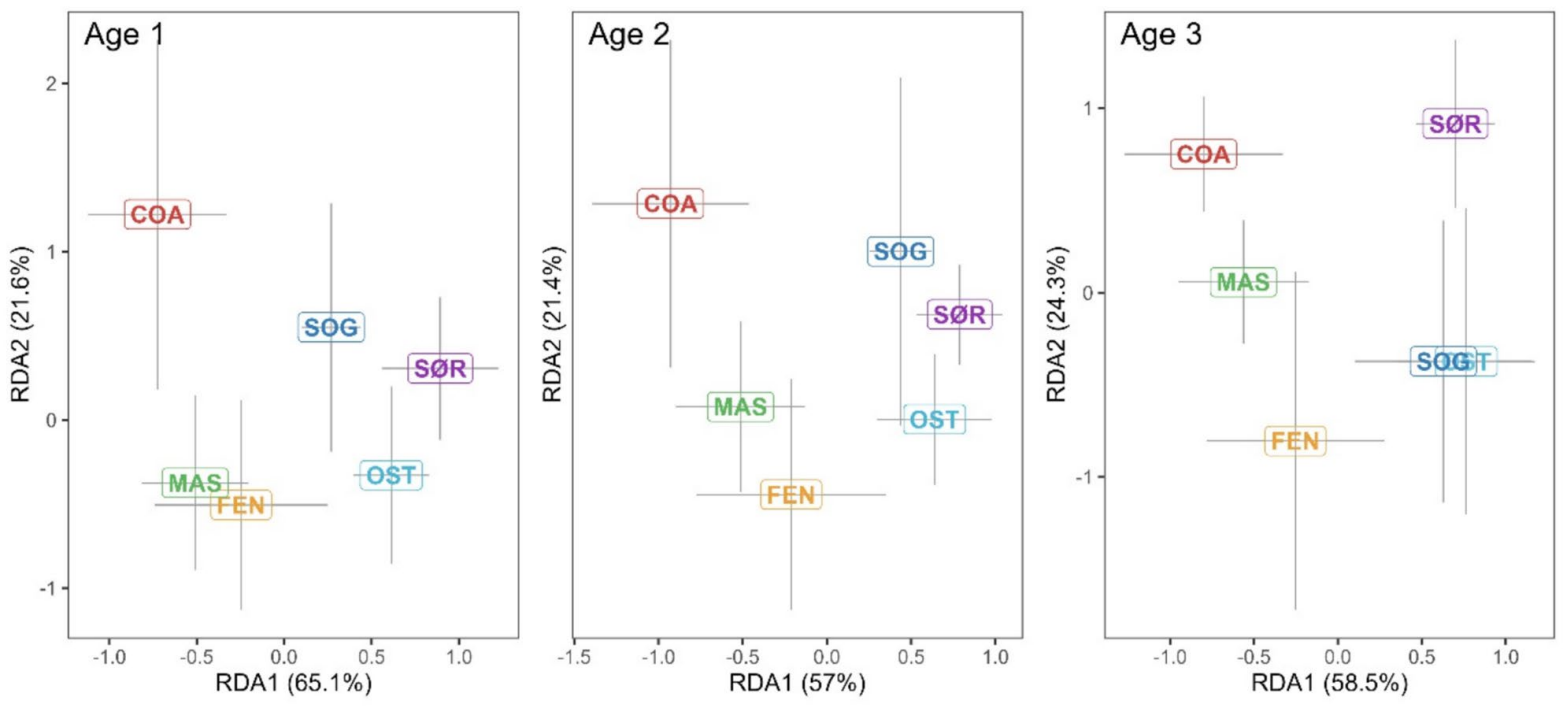
Redundancy Analysis (RDA) ordination diagrams representing variations in otolith trace element composition of *B. glaciale* between selected locations across the west Norwegian coast. Separate RDAs were conducted for otolith increments formed at ages 1, 2, and 3.

Consistent patterns of variation across the six sampling sites for otolith increments formed at ages 1 to 3 were revealed by the RDA-based ordinations of trace element concentrations (Fig. 5). Along the first CC (accounting for 57-66% of the explained variance), the three sites located closer to the outer coastline (COA, FEN, MAS) predominantly exhibit negative values. In contrast, the more inland sites (OST, SOG, SØR) show positive values. On the second CC (accounting for 21-24% of the explained variance), the northernmost samples (COA and SOG) tend to display higher scores, except for age 3, where the SOG centroid shifts closer to OST. Examining this axis reveals an increasing distance with age within each pair of fjords separated by sills (FEN-MAS and OST-SØR). The contributions of the sampled trace elements to the first two RDA axes are provided in Supplementary Fig. S2.

FEN and MAS show again the greatest similarity in trace element composition, as supported by the hierarchical cluster analysis (Supplementary Fig. S3). COA and SOG display the largest differences, consistent with what was observed for otolith shape. At the same time, the southern fjords exhibit closer relationships overall.

Trace element composition varies significantly among most sampling sites, as indicated by pairwise comparisons of the canonical scores (PERMANOVA, BH α = 0.05; Table 4), with FEN and MAS being the only pair without substantial differences across all analysed age classes.

**Table 4:** Statistical significance (after Benjamini–Hochberg correction) of pairwise comparisons based on Permutational Analysis of Variance (PERMANOVA, Euclidean distances with 2000 permutations) representing differences in otolith trace element composition of *B. glaciale* sampled in six sites along the west Norwegian coast.

		COA	FEN	MAS	OST	SOG
**Age 1**	FEN	0.028 *				
	MAS	0.030 *	0.755			
	OST	0.001 ***	0.048 *	0.001 ***		
	SOG	0.032 *	0.097 .	0.002 **	0.100	
	SØR	0.001 ***	0.002 **	0.001 ***	0.016 *	0.003 **
**Age 2**	FEN	0.013 *				
	MAS	0.077 .	0.367			
	OST	0.001 ***	0.044 *	0.001 ***		
	SOG	0.015 *	0.009 **	0.001 ***	0.047 *	
	SØR	0.001 ***	0.001 ***	0.001 ***	0.001 ***	0.001 ***
**Age 3**	FEN	0.027 *				
	MAS	0.229	0.250			
	OST	0.002 **	0.047 *	0.003 **		
	SOG	0.007 **	0.039 *	0.003 **	0.183	
	SØR	0.003 **	0.002 ***	0.001 ***	0.001 ***	0.003 **

The analysis was performed separately for increments formed during age 1, 2 and 3. Bold values highlight statistical significance (p < 0.05). Symbols: .<0.10, *<0.05; **<0.01, ***<0.001.

Geographic distance showed a weak correlation with trace element variation, just above the significance level in otolith increments formed at age 1 (Mantel tests, p = 0.069) and 2 (p = 0.057) (Supplementary Fig. S4). A positive and significant correlation emerged at age 3 (r = 0.68, p = 0.043).

The analysis conducted on a subset of data including only fish from the cohorts FEN 2016, FEN 2017, MAS 2016, and SØR 2016 revealed patterns of variation consistent with those observed using the full set of cohorts: the chemical signature recorded in otolith increments from FEN and MAS at ages 1 to 3 did not significantly differ within or across cohorts, while both fjords differed from SØR. Similarly, a stable signal was detected in FEN across the two cohorts (2016 and 2017), regardless of age class. (Supplementary Table S5).

## Discussion

To our knowledge, this study presents the first record of regional variation in otolith shape and trace element composition in the glacier lanternfish, *Benthosema glaciale*. Both methods suggest fine-scale population structuring consistent with findings outlined by previous genetic studies^15,38,39^.

The hypothesis that sills shallower than 130 m induce differentiation between basins^38^, however, appears unsupported by our data: we found no significant differences between individuals from Fensfjord and Masfjord (separated by a 75 m deep sill), while the coastal site and the deep-silled Fensfjord, as well as the neighbouring Osterfjord and Sørfjord (sill depth 160 m), showed heterogeneity in otolith shape and the analysed trace elements. The analysis of trace element compositions further illustrated a complete overlap between Fensfjord and Masfjord in the multivariate space for increments formed at age 1, followed by increasing divergence at older ages. These results imply that, for *B. glaciale*, other segregation mechanisms play a more significant role than shallow topographic structures.

### Fjord-coast differentiation in otolith shape and microchemistry

Our results support the existence of fjord-associated population components of *B. glaciale* that are partially isolated from those inhabiting adjacent coastal waters. Excluding the samples from Sogndalsfjord—a basin geographically distant from the other sampling sites and constrained by a 25-meter-deep sill—the most pronounced differences in the data were observed between fjords and the coastal site. The interplay between geographic and hydrographic boundaries is a well-established factor shaping the structure of marine populations, as demonstrated by studies on other small pelagic species using genetic or phenotypic tools^76,77^. For *B. glaciale* in our study region, evidence of partial structuring was also observed in their growth patterns, which were found to differ between fjord and open water populations^15^. These observations are consistent with broader ecological and evolutionary theories, implying that partial isolation and local environmental factors play crucial roles in shaping genetic diversity and phenotypic traits. As climate change becomes a serious issue in coastal areas worldwide^78^ these basins might serve as hotspots of differentiation, enhancing the adaptive potential of local subpopulations^79^.

### Connectivity across adjacent basins

In agreement with the genetic study by Suneetha and Salvanes^39^, we found Masfjord to be significantly different from the coastal site in both otolith shape and microchemistry. The 75 m deep sill isolating Masfjord from the large and deep-silled Fensfjord (which opens onto the coast) has been hypothesised to constitute the main barrier to gene flow^39^, considering the deep distribution of adult *B. glaciale* during most of the day. Divergence between Masfjord and open waters has also been recorded for the deep-dwelling *Coryphaenoides rupestris*^80^ and for the other abundant mesopelagic fish in the region, *Maurolicus muelleri*^41^. Contrary to our expectations, however, the otolith shape and microchemical signature of *B. glaciale* caught in Masfjord did not stop at the sill, but extended into Fensfjord. Here, we propose several hypotheses that can explain the lack of divergence we observed between fish from these two neighbouring basins.

*Benthosema glaciale* larvae in the preflexion stage prey mainly on copepod eggs and nauplii, while later stages feed on larger calanoid copepodites occurring deeper^81^. Passive transport of early life stages represents a primary dispersal pathway for a species performing limited horizontal movements^82^. In the Mediterranean and Celtic seas, larvae of *B. glaciale* were observed to distribute at depths of 20 to 100 m, feeding throughout the day and adjusting their depth based on prey availability rather than diel light cycles^81^. Due to the proximity of coastal waters, displacement of the advective intermediate layers between Fensfjord and Masfjord occurs periodically, with oscillations induced by wind frequency and strength along the Norwegian coast^12,83^. In light of this, a significant exchange of early-stage larvae between the two basins is a possibility.

Post-metamorphosis life stages of *B. glaciale* in Masfjord are distributed mostly below the sill depth during daytime. At night, they ascend to the upper layers of the water column including the first 100 m, as observed over the deep basin of the fjord^50^. Due to their size-related vertical distribution^46^, smaller, younger fish are found closer to the surface compared to larger adults. In our annual survey in the fjords, in which we employ a Multisampler, it is not uncommon to catch smaller *B. glaciale* together with *M. muelleri* in the upper 100 m over the deep basin of the two fjords at night. Observations made by Kaartvedt and colleagues^84^ reported *B. glaciale* as mostly inactive and drifting with weak tidal currents, “essentially acting as plankton” and only occasionally swimming in small, steep bursts to adjust their vertical position in the water column. Although Aksnes and colleagues^85^ reported only a few *M. muelleri* caught over the sill during a pelagic trawl survey in 1989, it is not impossible for individuals feeding above sill depth to be passively transported during in- or outflow events.

Although otoliths from Fensfjord and Masfjord did not differ significantly in trace element composition at ages 1–3, the microchemistry-based RDA suggests a slight tendency toward increasing divergence with age between neighbouring fjords. This pattern is weak and should be interpreted cautiously, but it may reflect modest environmental contrasts between the fjords particularly in deeper waters, where Masfjord is characterised by lower deep basin oxygen and more restricted exchange due to its shallow sill (Supplementary Fig. S5).

Overall, the strong overlap in otolith shape between the two fjords and the close similarity in trace element composition suggest that the sill is not the main factor driving isolation in Masfjord. While this barrier is likely to contribute to some extent to the segregation of larger individuals, and advection mechanisms can be expected to be more relevant for planktonic life stages than juvenile fish (which undergo diel vertical migrations and can swim actively), our results indicate that *B. glaciale* in Fensfjord and Masfjord do not represent two separate units.

Recent studies revealed genetic heterogeneity in both *B. glaciale*^38^ and in *Melanogrammus aeglefinus* from Osterfjord and other fjords and coastal sites^86^. The relative isolation of Osterfjord is also evident in our results, as indicated by both otolith shape and chemical signals of *B. glaciale*. Our samples, however, also included Sørfjord, a neighbouring basin of Osterfjord, for which genetic studies are lacking. The lower but substantial differentiation found in our data between the Osterfjord and Sørfjord was unexpected, especially since a 160 m deep passage separates the fjords—thus considerably deeper than the sill between Fensfjord and Masfjord, where we saw no differences.

Parasitic infections have been used as natural tags at various spatial scales in several pelagic fish species^87^. Observations from a recent study on parasitic copepod infection in *B. glaciale*^88^ show consistent patterns in cross-fjord connectivity to our results, reporting a higher prevalence in adult fish from Masfjord and Osterfjord compared to Fensfjord and Sørfjord. Considering our results, the differences in parasite prevalence between adjacent fjords could also be attributed to the retention of adult individuals within basins. Moreover, advective strength dampens markedly with distance from the coast^83^. It is therefore reasonable to expect reduced water exchange in and between basins further away from the coast (such as the Osterfjord/Sørfjord system), given their relatively landlocked nature^51^. The increased volume available for exchange over the deeper sill between Osterfjord and Sørfjord might therefore be offset by retention caused by local oceanographic dynamics.

Based on our results, we suggest that oceanographic connectivity due to current circulation affecting the dispersal of early life stages, rather than shallow topographic features, is the main driver structuring coastal populations in small mesopelagic fish. Similar mechanisms have been observed for the myctophid *Electrona antartica* in the open waters of the Southern Ocean^89^, and in other pelagic species such as herring in the Baltic Sea^90^. This may also account for the weak significance of the relationship between otolith shape/trace element variation and geographic distance among our sampling sites (Supplementary Fig. S1, S4). In this regard, the stronger relationship between geographic distance and chemical signature for increments formed during age 3 (i.e. after sexual maturation, when individuals distribute deeper) compared to that of juveniles might be interpreted as further evidence for this dynamic.

Despite expectations of homogeneity in otolith shape and microchemistry between the deep-silled Fensfjord and the coastal site, our results revealed significant differences in these traits. Strong winds from the north are common along the Norwegian coast between May and August, when early life stages of *B. glaciale* are present in the fjords^51,91^. Northerly winds flush the less saline surface layers out of the fjords, generating coastal upwelling and a corresponding inflow of deep Atlantic water below^12^. It is also important to recognise that, even when mixing occurs during early life stages, environmental differences experienced in later life can dampen early-life phenotypic signals, as otolith morphology is sensitive to local environmental conditions and can therefore shift during settlement and adulthood^92^. In this study, we therefore interpret our results as a relative measure of connectivity between the sampled sites, rather than an absolute measure of isolation.

### Further considerations on otolith shape and microchemistry

Otolith shape variations within species have been demonstrated to be under genetic and environmental regulation. Data exploration on our otolith shape coefficients (as described by Libungan & Pálsson^66^) revealed that most of the between-group variation in our study area occurs along the dorsal and ventral edge of the otolith. In *B. glaciale*, these regions correspond to the portion where the postdorsal angle and ventral spikes tend to form (for a detailed description of otoliths of *B. glaciale* and related nomenclature, see Hoedemakers et al.^93^). These features can be more or less pronounced across individuals and, in the case of the ventral spines, range in number from 0 to 4^93^. While some of these characteristics appear to relate to age (as with most fishes, adult otoliths of *B. glaciale* become more sculpted with age), a visual assessment of our images from individuals aged 2-5 does not show any obvious ontogenetic patterns. In a study of variation in otolith shape of Baltic Sea herring (*Clupea harengus*), Berg and colleagues^30^ found a strong genetic signal on the first axis of their Canonical Analysis of Principal Coordinates. In contrast, the second axis appeared to capture the salinity gradient between rearing environments. Temperature^27^, dissolved oxygen concentration^28^ and diet^29^ have also been observed to affect otolith growth patterns. Temperature, oxygen and salinity profiles exemplifying local conditions at our sampling sites are given in Supplementary Fig. S5. A similar pattern to what was reported in^30^ can be identified in our otolith shape data, with a signal of reproductive isolation captured by the first RDA axis and a latitudinal/environmental signal on the second. These broad patterns appear consistent with a framework of isolation by distance and water exchange as the principal driver for the spatial structure captured by our data.

Variations in fish otolith microchemistry reflect both environmental influences and physiological responses^24^. Our multivariate analysis of trace element composition indicates that otoliths from Sogndalsfjord and Sørfjord exhibit higher levels of iron (Fe) and manganese (Mn) (Supplementary Fig. S2), suggesting higher potential inputs from terrestrial runoff^94^. This is consistent with topographic considerations and the relative size of the catchment basins of these fjords (Supplementary Table S1). Mn is notably low in seawater^95^. Its increased presence, especially in the otoliths of fish aged 1 and 2, might imply a link to riverine sources or coastal wetlands to inland fjord nursery areas for juvenile *B. glaciale* in the region. Furthermore, our data demonstrates a close association between Fensfjord and Masfjord within the microchemistry ordination space for increments deposited at age 1. However, as fish age, a noticeable divergence occurs along the directions in which the phosphorus (P) and barium (Ba) vectors contribute (Supplementary Fig. S2). As P regulation in otoliths is tightly controlled physiologically and assumed to relate to feeding or growth rates^24^, this shift could reflect a prevalence of shared resources early in life and reduced ecological interactions between the two basins at greater depths. Similarly, Ba has been shown to increase with depth below the thermocline^36,96^. The increased difference in Ba with age between the two fjords could suggest a deeper distribution of adult fish in Fensfjord compared to Masfjord, where light penetration is reduced, and the light comfort zone of *B. glaciale* could be encountered at lower depths.

The main objective of our investigation was to test the potential of otolith shape and trace element composition to characterise fine-scale population connectivity in a myctophid species. For a deeper understanding of myctophids’ life histories and ecological requirements, further studies are necessary to elucidate spatial, temporal and ontogenetic variations in otolith features. Determining how these characters vary across different habitats or life stages would provide information needed to predict how ongoing environmental change, such as shifts in temperature, oxygen or wind regime, may affect population dynamics and connectivity.

## Conclusions

The analysis of fish otoliths is a fundamental aspect of fisheries research, enabling the reconstruction of individual growth trajectories and population structure. Here, the use of two independent approaches–shape analysis and microchemistry–provides novel insights into the population structure of the glacier lanternfish *Benthosema glaciale* along the west Norwegian coast. Our data support the existence of semi-isolated population components within a relatively short geographic distance and emphasise the influence of local environmental factors and oceanographic dynamics in shaping mesopelagic fish population units. This work highlights the importance of ongoing investigations into the population structure and dynamics of *B. glaciale*, and provides the first evidence of the effectiveness of otolith morphometrics and elemental composition in addressing ecological questions in this highly abundant species. By applying complementary multidisciplinary approaches, future research should aim to develop a comprehensive understanding of connectivity patterns and potential stock boundaries, ultimately supporting sustainable management and conservation efforts for this and other mesopelagic species.

## Supplementary Information

Supplementary Information 1.

## Data Availability

The raw data used in this study are available upon request to the corresponding author.
