# Supplementary material for: Otolith shape and microchemistry reveal fine-scale population connectivity in the myctophid Benthosema glaciale (Reinhardt, 1837) along a complex seascape
Source: Sci Rep. 2026 Apr 1;16:18982. doi: 10.1038/s41598-026-46216-3 (PMC13276378; doi:10.1038/s41598-026-46216-3)
Supplement: Supplementary file 1 — Supplementary Information 1. [file 41598_2026_46216_MOESM1_ESM.docx]

Supplementary information and supplementary material for:

**Otolith shape and microchemistry reveal fine-scale population connectivity in the myctophid *Benthosema glaciale* (Reinhardt, 1837) along a complex seascape**

**Francesco Saltalamacchia ^1, 2, *^, Natalya Gallo ^1, 2, 3^, Karin Limburg ^4, 5^, Arild Folkvord ^1, 6^ & Anne Gro Vea Salvanes ^1, 2^**

^1^ Department of Biological Sciences, University of Bergen, Bergen, Norway

^2^ Bjerknes Centre for Climate Research, Bergen, Norway

^3^ Norwegian Research Centre (NORCE), Bergen, Norway

^4^ Department of Environmental Biology, State University of New York College of Environmental Science and Forestry, Syracuse (New York), USA

^5^ Department of Aquatic Resources, Swedish University of Agricultural Sciences, Uppsala, Sweden

^6^ Institute of Marine Research (IMR), Bergen, Norway

^*^ E-mail: francesco.saltalamacchia@uib.no

# Supplementary information

**Supplementary information S1**

First, we applied a Redundancy Analysis (RDA) from the *vegan* package ^1^, constrained by sampling site and side (left or right), to the matrix of shape coefficients. This allowed the compression of the multivariate space into fewer dimensions (canonical components, CCs) while avoiding collinearity. To test for shape variation across the study region while considering potential bilateral asymmetry, we fed the matrix of CCs into a set of nested linear multivariate mixed-effect models, with SL (to check for any residual effects of body size), side (left-right) and sampling site (N = 6) as fixed predictors. This type of model is well-suited for continuous response variables with mixed negative and positive values (such as canonical scores) and repeated measurements (two otoliths from the same individual). All models were fitted using the *nlme* package v. 3.1-168 ^2^, including a random intercept for each individual (to account for autocorrelation within otolith pairs and individual variability in shape), and allowing the variance to differ between sites. All continuous variables (the CC outcomes and the SL predictor) were centred to avoid issues with numerical estimation and aid model interpretation. Type II likelihood ratio tests on nested models were implemented through the *car* package v. 3.1.2 ^3^ to test for the significance of predictors. Model validation was performed on the best model using the *ggResidpanel* package v. 0.3.0 ^4^ to evaluate the normality and homoscedasticity of the residuals, as well as the relationship between Pearson residuals and both fitted values and observation number. Similarly, the normality of the random effects was examined through a quantile-quantile plot.

**Supplementary information S2**

The laser fluence was 0.81 J·cm^-2^ for pre-ablation and 1.62 J·cm^-2^ for ablation, with a spot diameter of 50 μm and a travel speed of 3 μm·s^-1^. A NIST612 calibration material (U.S. National Institute of Standards and Technology) was sampled at the beginning of each session to optimise the daily performance of the system. Before each transect, the machine performed a 30-second gas blank acquisition. To calibrate the conversion from counts per second (cps) to parts per million (ppm) and account for variations in detection sensitivity during each session (“machine drift”), every series of 7-10 otolith ablations was bracketed by acquisitions from a micro-analytical phosphate standard (MAPS4, U.S. Geological Survey). As MAPS materials are slightly softer than otoliths, ablation was performed using a laser fluence of 2.03 J·cm^-2^ ^5^, with a spot diameter of 50 μm and a speed of 4 μm·s^-1^. Each laser transect produced a matrix containing a timestamp for each ablation point and separate columns for analyte concentrations (cps).

**Supplementary information S3**

The blank signal recorded before each transect was used for interference (background) correction through the *autosmooth spline* function implemented in Iolite, as well as for calculating the limits of detection (LODs). Data reduction was performed using the software’s semi-quantitative reduction scheme. This approach estimates the concentration of each analyte in ppm at any point along the otolith transect by comparing the background-corrected cps with those detected for the reference material, where the ppm concentration is known. Each value is adjusted based on the spline extrapolated from the regularly interspersed reference material replicates to account for machine drift.

Some trace elements occur in fish otoliths in relatively small concentrations. To discriminate whether a low value was real or random noise, limits of detection (LODs) were calculated in Iolite for each analyte and sample as three times the standard deviation of the average concentration measured during the preceding blank phase ^6^. Further processing and analysis were performed in R. All below-LOD measurements were marked for subsequent correction. Among the sampled analytes, eight (Li, Na, Mg, P, Ca, Fe, Sr, Ba) had, on average, less than 1% below-LOD measurements per transect; four (B, Mn, Cu, Zn) had between 7 and 14%, and three (Pb, Co, Cr) had more than 20% (24, 45, 78% respectively). To ensure representative transects, we chose to discard the last three. All the other elements were retained for further analysis.

During ablation, the laser may occasionally dislodge larger particles or traverse small cracks in the section. Due to this, major spikes and dips in analyte concentrations are not uncommon in LA-ICP-MS data. These artificial anomalies are relatively easy to detect, as they occur on a different magnitude compared to the typical variation recorded along the transect, are not palindromic (i.e., do not occur symmetrically on both sides of the core), and are usually formed by a single measurement point. Following a visual examination of the data for all otoliths and analytes, we addressed the issue by considering as outliers any data points that fell, within each transect, beyond 1.5 interquantile ranges below the 10^th^ or above the 90^th^ percentile. Additionally, for a point to be classified as an outlier, neither the preceding nor the following data points must exceed the identified thresholds. All transects underwent a second visual inspection, and a few implausible measurements not detected by the defined thresholds were manually labelled as outliers.

# Supplementary material

**Supplementary Table S1** Geographic information about the sites sampled for the study of otolith shape and microchemistry of Benthosema glaciale along the Norwegian west coast. Distance from the coast was measured as the shortest distance from the mouth of each fjord to the outer coast. Fjord length and surface area were measured in ImageJ from the sill to the end of the main fjord axis. Watershed area for each fjord was calculated from the REGINE register of catchments compiled by the Norwegian Water Resources and Energy Directorate (https://temakart.nve.no/tema/nedborfelt, accessed on 03.06.2025). NB: Even though Masfjord branches out of Fensfjord, the watershed areas of the two fjords were kept separated for the purpose of this comparison.

| **Site** | **Abbreviation** | **Distance from coast (km)** | **Fjord length (km)** | **Fjord surface (km^2^)** | **Watershed area (km^2^)** | **Watershed to surface area** |
| --- | --- | --- | --- | --- | --- | --- |
| Coastal | COA | 0 | - | - | 58.3 | - |
| Fensfjord | FEN | 33.3 | 30 | 152.3 | 223.6 | 1.5 |
| Masfjord | MAS | 50.2 | 24 | 28.5 | 597.7 | 21.0 |
| Osterfjord | OST | 61.5 | 27 | 72.4 | 913.8 | 12.6 |
| Sogndalsfjord | SOG | 143.2 | 21 | 22.6 | 769.5 | 34.0 |
| Sørfjord | SØR | 79.8 | 30 | 53.5 | 2656.2 | 49.6 |

**Supplementary Table S2** Results of the multivariate mixed-effect model on otolith shape coefficients of B. glaciale. Explanatory variables: SL, standard length (mm); Side, left or right otolith; Site: sampling sites. The corresponding χ² statistic, degrees of freedom (df), and p-value (p) for the Type II test are provided for each variable and interaction. Symbols: .<0.10, *<0.05; **<0.01, ***<0.001.

|  | ***χ^2^*** | **Df** | **p** |
| --- | --- | --- | --- |
| SL | 2.389 | 1 | 0.122 |
| Side | 2042.687 | 1 | **0.001 ***** |
| Site | 82.006 | 5 | **0.001 ***** |
| Side:site | 40.390 | 5 | **0.001 ***** |

**Supplementary Table S3** Statistical significance (after Benjamini–Hochberg correction) of pairwise comparisons based on Permutational Analysis of Variance (PERMANOVA, Euclidean distances with 2000 permutations) representing variation in trace element composition between left and right otoliths of *B. glaciale* in three of the six sampling sites*.*  At each site, sample size for left and right otoliths (n L-R) and statistical significance (p) are provided separately for increments formed at ages 1-3. NB: Left and right otoliths in this analysis originate from different individuals.

|  | Age 1 | | Age 2 | | Age 3 | |
| --- | --- | --- | --- | --- | --- | --- |
|  | **n L-R** | **p** | **n L-R** | **p** | **n L-R** | **p** |
| FEN | 6-5 | 0.638 | 6-5 | 0.979 | 5-5 | 0.223 |
| MAS | 6-7 | 0.119 | 5-7 | 0.623 | 5-7 | 0.380 |
| SØR | 4-4 | 0.747 | 4-4 | 0.915 | 4-4 | 0.256 |

**Supplementary Table S4** Number of individuals per cohort used in the analysis of trace element variation of *B. glaciale* across six sites on the Norwegian west coast. Cohorts were pooled within each site.

| **Cohort** | **2013** | **2014** | **2015** | **2016** | **2017** | **2018** | **2019** |
| --- | --- | --- | --- | --- | --- | --- | --- |
| COA | 1 | 1 | 1 | 2 | 1 |  |  |
| FEN |  |  | 2 | 5 | 4 |  |  |
| MAS |  |  | 3 | 7 | 3 |  |  |
| OST |  | 2 |  | 2 | 3 | 1 |  |
| SOG |  |  |  | 1 | 1 | 3 | 1 |
| SØR |  |  | 3 | 5 |  |  |  |

**Supplementary Table S5** Statistical significance (after Benjamini–Hochberg correction) of pairwise comparisons based on Permutational Analysis of Variance (PERMANOVA, Euclidean distances with 2000 permutations) representing differences in trace element composition in otolith increments of B. glaciale formed at ages 1-3. The comparison was performed on data from the 2016 cohort from three of the six sampling sites. The 2017 FEN cohort is also included as a check for within-site differences across cohorts. Sample size: FEN 2016 (n = 5), FEN 2017 (4), MAS 2016 (7), SØR 2016 (5). At age 3, the sample size for FEN 2016 is 4. Symbols: .<0.10, *<0.05; **<0.01, ***<0.001.

|  |  | **FEN_2016** | **FEN_2017** | **MAS_2016** |
| --- | --- | --- | --- | --- |
| **Age 1** | FEN_2017 | 0.323 |  |  |
|  | MAS_2016 | 0.184 | 0.502 |  |
|  | SØR_2016 | **0.011 *** | **0.008 **** | **0.001 ***** |
| **Age 2** | FEN_2017 | 0.141 |  |  |
|  | MAS_2016 | 0.100 | 0.234 |  |
|  | SØR_2016 | **0.022 *** | **0.008 **** | **0.001 ***** |
| **Age 3** | FEN_2017 | 0.709 |  |  |
|  | MAS_2016 | 0.224 | 0.464 |  |
|  | SØR_2016 | **0.015 *** | **0.030 *** | **0.009 **** |


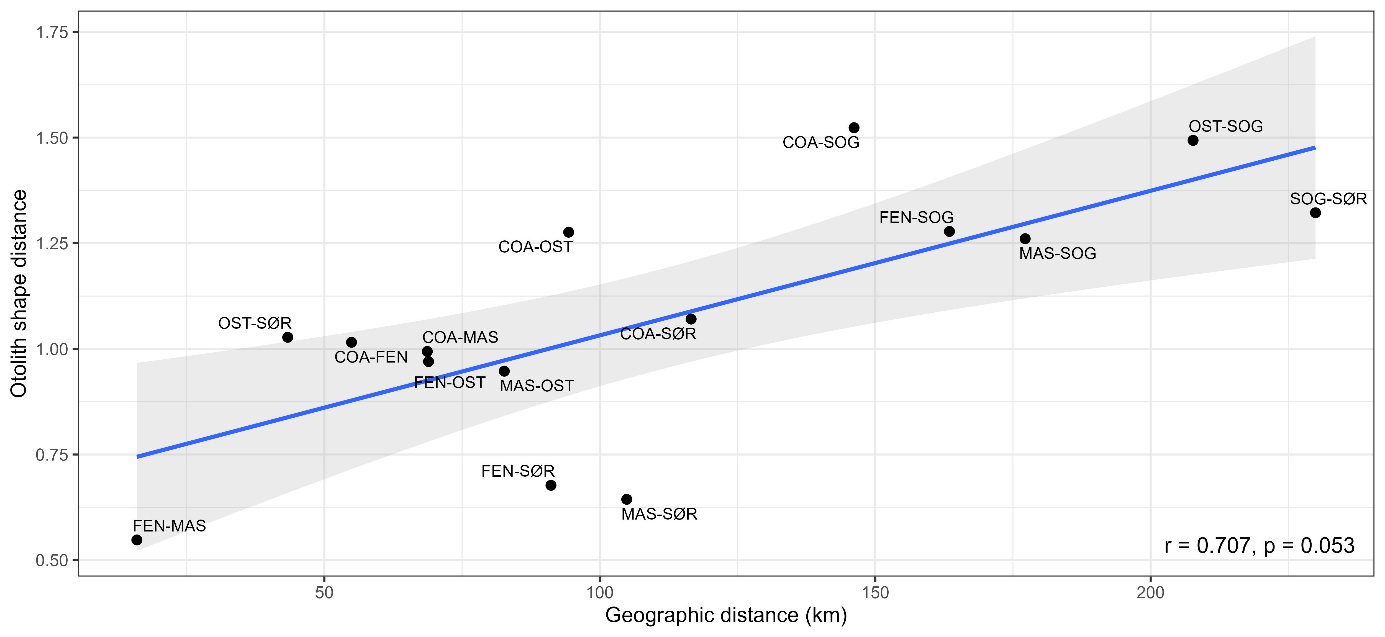


**Supplementary Fig. S1** Pairwise left-side otolith shape distances of B. glaciale against geographic distances (N = 6 sites). Results of the Mantel correlation tests are given as Spearman correlation coefficient and p-value under 2000 permutations. Sites: COA (Coastal), FEN (Fensfjord), MAS (Masfjord), OST (Osterfjord), SOG (Sogndalsfjord), SØR (Sørfjord).


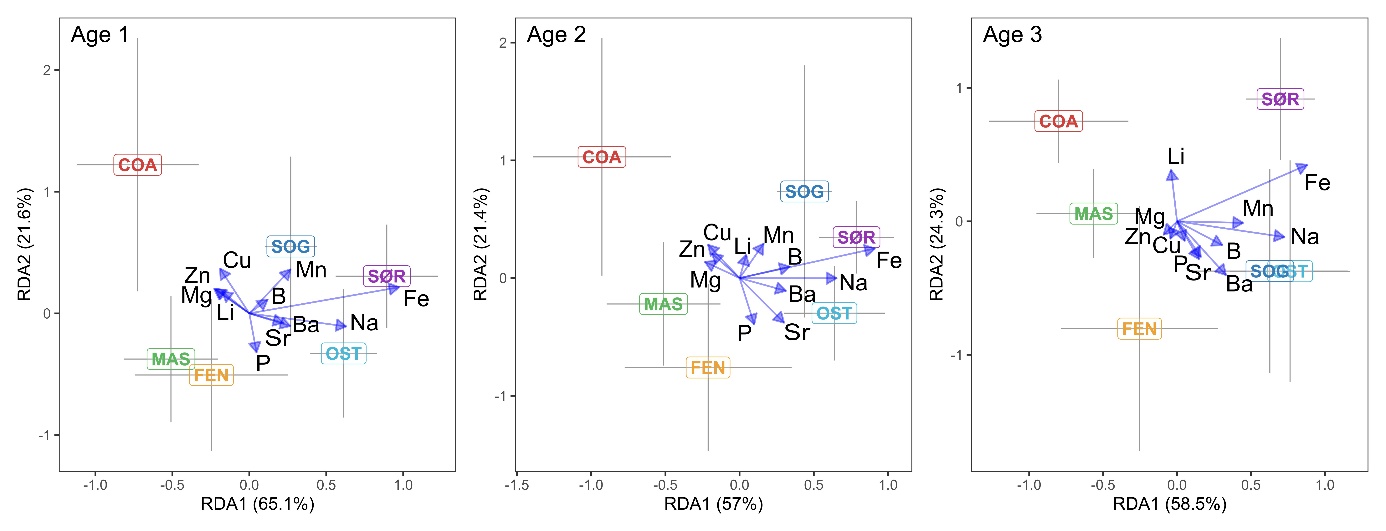


**Supplementary Fig. S2** Redundancy Analysis (RDA) ordination diagrams as described in Fig. 5, with overimposed information on the contribution of each sampled trace element along the first two canonical axes. Each arrow’s direction and length represent its direction of influence and relative importance.


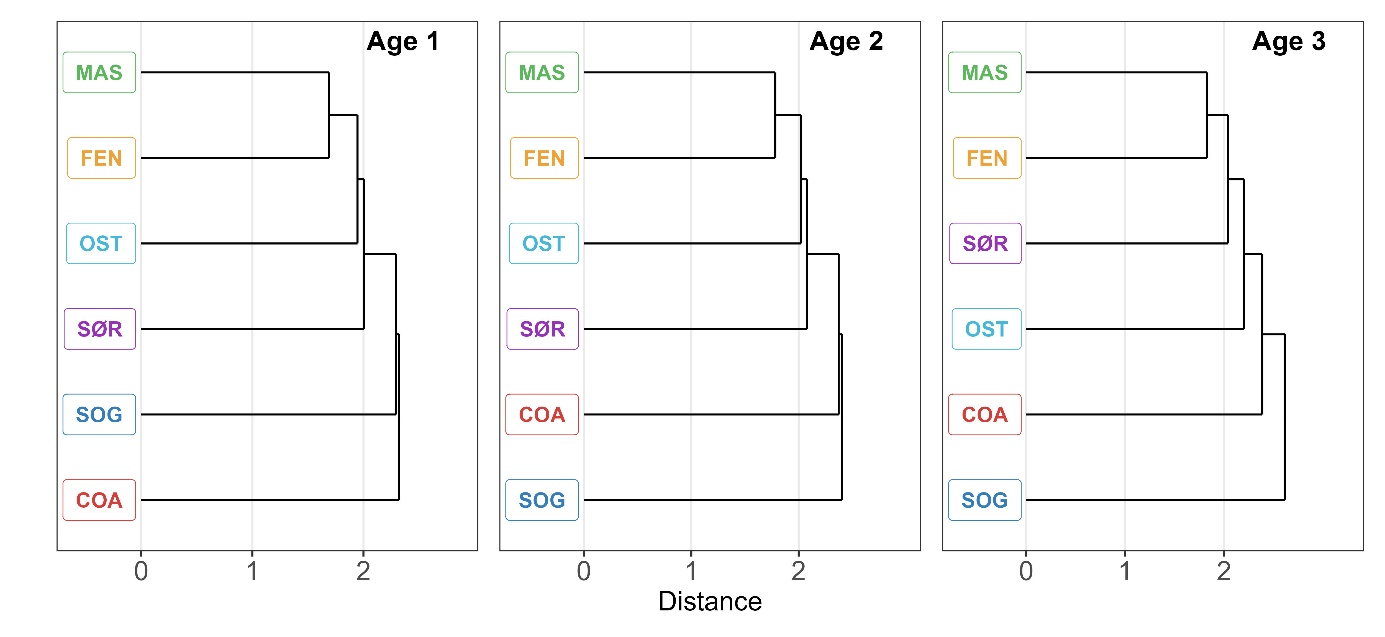


**Supplementary Fig. S3** Dendrogram of otolith trace element relationships of B. glaciale across six sampling sites along the Norwegian west coast, generated by Ward’s hierarchical agglomerative algorithm using squared Euclidean distances. The analysis was conducted separately on increments formed during ages 1, 2 and 3.


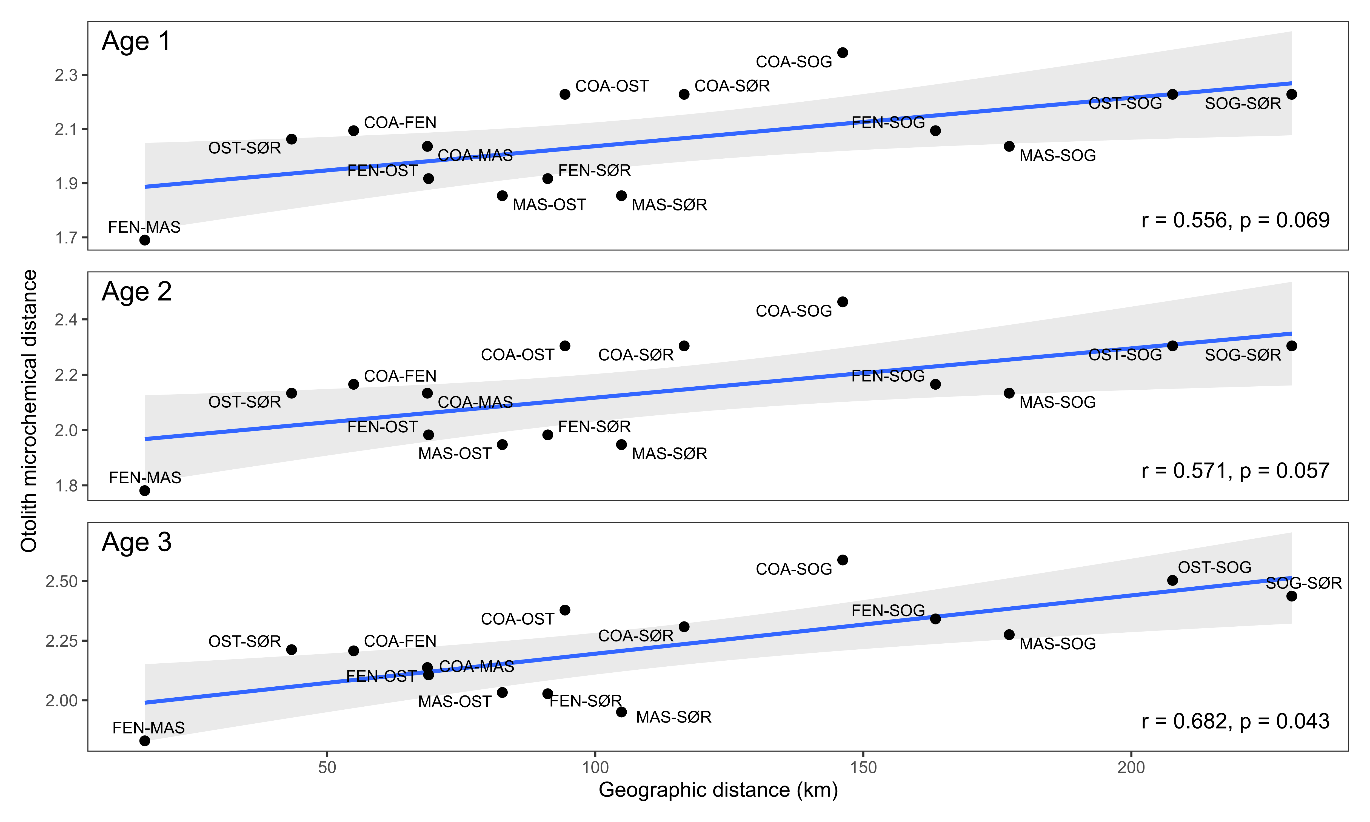


**Supplementary Fig. S4** Pairwise otolith trace element concentration distances of B. glaciale against geographic distance (N = 6 sites). Results of the Mantel correlation tests are given as Spearman correlation coefficient and p-value under 2000 permutations. Sites: COA (Coastal), FEN (Fensfjord), MAS (Masfjord), OST (Osterfjord), SOG (Sogndalsfjord), SØR (Sørfjord).


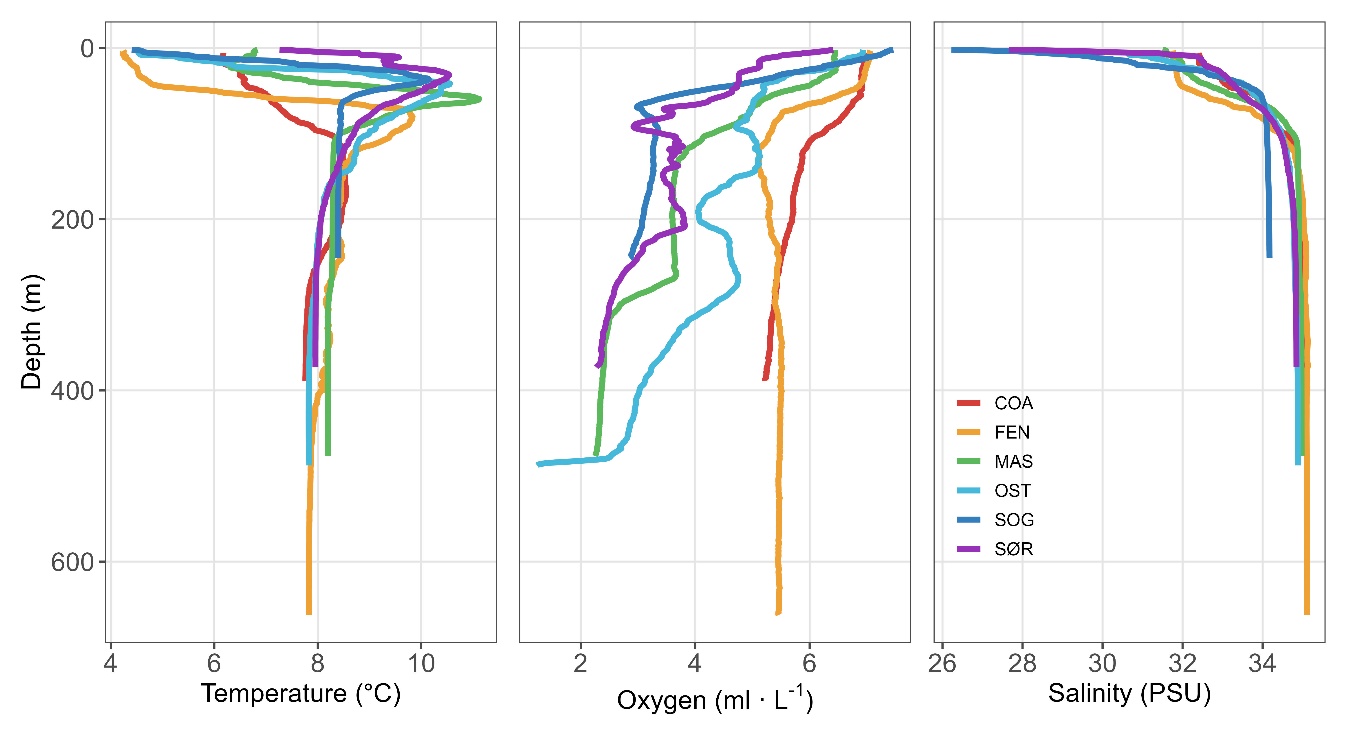


**Supplementary Fig. S5** Temperature, oxygen and salinity profiles at the six sampling sites. Environmental parameters were collected with a Seabird CTD in February 2021 (FEN, MAS, OST, SØR) and February 2022 (COA, SOG).

# References

1. Oksanen, J. *et al.* vegan: Community Ecology Package. (2022).

2. Pinheiro, J. C. & Bates, D. M. nlme: Linear and Nonlinear Mixed Effects Models. (2025).

3. Fox, J. & Weisberg, S. An R companion to applied regression. (2019).

4. Katherine, G. & Kathleen, R. ggResidpanel: Panels and Interactive Versions of Diagnostic Plots using ‘ggplot2’. (2019).

5. Limburg, K. E., Heimbrand, Y. & Kuliński, K. Marked recent declines in boron in Baltic Sea cod otoliths – a bellwether of incipient acidification in a vast hypoxic system? *Biogeosciences* **20**, 4751–4760 (2023).

6. Longerich, H. P., Jackson, S. E. & Günther, D. Inter-laboratory note. Laser ablation inductively coupled plasma mass spectrometric transient signal data acquisition and analyte concentration calculation. *J. Anal. At. Spectrom.* **11**, 899–904 (1996).
